# Supplementary material for: A non-catalytic herpesviral protein reconfigures ERK-RSK signaling by targeting kinase docking systems in the host
Source: Nat Commun. 2022 Jan 25;13:472. doi: 10.1038/s41467-022-28109-x (PMC8789800; doi:10.1038/s41467-022-28109-x)
Supplement: Supplementary file 1 — Supplementary Information [file 41467_2022_28109_MOESM1_ESM.pdf]

Supplementary Information

A non-catalytic herpesviral protein reconfigures ERK-RSK signaling by targeting kinase docking systems in the host

Anita Alexa[1], Péter Sok[1], Fridolin Gross[2], Krisztián Albert[1], Evan Kobori[3], Ádám L. Póti[1], Gergő, Gógl[1], Isabel Bento[4], Ersheng Kuang[5], Susan S. Taylor[6], Fanxiu Zhu[5], Andrea Ciliberto[2], Attila Reményi[1,*]

[1]Biomolecular Interactions Research Group, Institute of Organic Chemistry, Research Center for Natural Sciences, H-1117 Budapest, Hungary.

[2]IFOM, Istituto FIRC di Oncologia Molecolare, 20139 Milan, Italy

[3]Department of Chemistry and Biochemistry, University of California San Diego, San Diego, 9500 Gilman Drive, La Jolla, CA 92093-0654, USA.

[4]European Molecular Biology Laboratory, Hamburg, Germany

[5]Department of Biological Science, Florida State University, Tallahassee, FL 32306-4370, USA

[6]Department of Pharmacology, University of California San Diego, 9500 Gilman Drive, La Jolla, San Diego, CA 92093-0654, USA.

*Correspondence: remenyi.attila@ttk.hu

Supplementary Table 1. Crystallographic data collection statistics and structure refinement

	ppERK2-ORF45(27-40)	RSK2(NTK)-ORF45(16-76)
**Data collection**		
Space group	C 1 2 1	C 1 2 1
Cell dimensions		
a, b, c (Å)	210.09 41.15 51.70	170.29 88.62 160.35
α, β, γ (°)	90.00 92.35 90.00	90.00 95.42 90.00
Resolution range (Å)	47.12 -2.45 (2.55-2.45)	47.65-2.75 (2.82-2.75)
$CC_{1/2}$	0.997 (0.749)	0.998 (0.500)
R_{merge}†	0.116 (1.220)	0.111 (1.440)
$<I/\sigma(I)>$	10.1 (1.5)	11.5 (1.3)
Completeness (%)	99.3 (99.0)	100.0 (100.0)
Redundancy	8.5 (8.4)	7.1 (7.1)
No. reflections	140097 (15361)	437614 (32437)
**Refinement**		
$R_{work/}$ R_{free}	0.2352/0.2535	0.2087/0.2322
No. atoms	3208	16458
Protein	3122	16225
Ligand/ion	47	186
Solvent	39	47
B-factors (Å^2)	77.43	86.04
Protein	77.69	86.39
Ligand	67.68	60.83
Solvent	68.86	63.38
Ramachandran		
Favored (%)	97.08	97.85
Allowed (%)	2.92	1.85
Outliers (%)	0.0	0.30
Rotamer outliers (%)	0.91	1.04
R.m.s deviations		
Bond lengths (Å)	0.003	0.005
Bond angles (º)	0.783	0.899

†$R_{merge} = \Sigma_{hkl}\Sigma_i |I_i(hkl)-<I(hkl)>|/\Sigma_{hkl}\Sigma_i I_i(hkl)$

Supplementary Table 2. HDX-MS data collection and statistics
Source data are provided as Source data file.

Data Set	**RSK2**-ERK2	**RSK2**-ERK2-ORF45
HDX reaction details	20 mM HEPES, 150 mM NaCl, 1mM TCEP pD_{read} = 7.5, 25 °C	20 mM HEPES, 150 mM NaCl, 1mM TCEP pD_{read} = 7.5, 25 °C
HDX time course (min)	0.5, 1, 2, 5	0.5, 1, 2, 5
HDX control samples	Maximally-labeled Standard protein	Maximally-labeled Standard protein
Back-exchange (mean / IQR)	25% / 3%	
# of Peptides	261	261
Sequence coverage	99%	99%
Average peptide length / Redundancy	16.95/ 6.05	16.95/ 6.05
Replicates (biological or technical)	3 (technical)	3 (technical)
Repeatability	0.163 (average standard deviation)	0.163 (average standard deviation)
Significant differences in HDX (delta HDX > X D)	0.5 D (>99% CI)	

Supplementary Table 3. Starting and fitted parameters of the computational models

parameter	measured	SPR fit^	in vitro fit^	in cell fit^	unit	abbreviation
K_D(ERK:RSK)	2.5*	-	**2.4**	**2.6**	µM	KD_ER
K_D(ERK:pRSK)	12.5**	-	**12.2**	**13.1**	µM	KD_EpR
K_D(ORF45:RSK)	0.0012*	-	**0.0012**	**0.0012**	µM	KD_OR
K_D(ORF45:pRSK)	0.0012*	-	**0.0012**	**0.0012**	µM	KD_OpR
K_D(ERK:ORF45)	19.9*	-	**79.4**	**101.5**	µM	KD_EO
K_D(pERK:ORF45)	0.8*	-	**0.79**	**0.98**	µM	KD_pEO
K_D(MKK:ERK)	4.0*	-	**4.1**	**3.9**	µM	KD_EK
K_D(MKP:ERK)	-	-	4.6	**4.7**	µM	KD_EP
K_D(PP:RSK)	-	-	-	0.23	µM	KD_RP2
k_{off}(ERK:RSK)	10.0*	**13.3**	**13.5**	**16.2**	min^{-1}	koff_ER
k_{off}(ORF45:RSK)	0.05*	**0.044**	**0.044**	**0.043**	min^{-1}	koff_OR
k_{off}(ERK:ORF45)	-	-	570.3	**756.8**	min^{-1}	koff_EO
k_{off}(pERK:ORF45)	-	5.6	**5.7**	**7.3**	min^{-1}	koff_pEO
k_{off}(MKK:ERK)	-	-	20.0	**18.8**	min^{-1}	koff_EK
k_{off}(MKP:ERK)	-	-	34.7	**34.7**	min^{-1}	koff_EP
k_{off}(PP:RSK)	-	-	-	0.92	min^{-1}	koff_RP2
k_{cat}(MKK)	-		0.52	**0.56**	min^{-1}	kp_E
k_{cat}(ERK)	-		10.5	**10.2**	min^{-1}	kp_R
k_{cat}(bg)	-		-	0.0073	min^{-1}	kp_K_bg
k_{cat}(EGF)	-		-	0.48	min^{-1}	kp_K_egf
k_{cat}(MKP)	-		6.8	**6.8**	min^{-1}	kdp_E
k_{cat}(PP, RSK)	-		-	4.1	min^{-1}	kdp_R
k_{cat}(PP, MKK)	-		-	0.076	min^{-1}	kdp_K
a	-	0.087	**0.087**	**0.10**	µM	a
d	-	0.0027	**0.0026**	**0.0025**	-	d

***** This study (KD for MKK:ERK binding was measured by competitive FP assay; koff for ERK:RSK and ORF45:RSK was determined using the SPR BioEvaluation software, GE Healtcare)
** RSK has a phospho-switch region next to the MAPK docking motif and the binding affinity weakens (~ 5-fold) if this ERK regulated site is phosphorylated[1]
^ Parameters in normal or bold type were free or constrained, respectively.

Supplementary Table 4. List of oligonucleotides used for cloning

Primer name	Primer sequence (5'-3')
**Cloning and mutagenesis of ORF45**	
ORF45FL-HindIII-F (pEBTetD-Hygro)	TTAAGCAAGCTTGCCACCATGGCGATGTTTGTTCGCACG
ORF45FL-XhoI-R (pEBTetD-Puro/ Hygro)	CCAATCTCGAGATCCAGCCACGGCCAATTATAAGC
Orf45FL-NheI-F (pEBTetD-Puro)	AATTAAGCTAGCATGGCGATGTTTGTTCGCACGAGC
Orf45-16-HindIII-F	CAGAGAAGCTTCCACCATGCGTATGCTGCCGATCGAAGG
ORFpep60CCys-NotI-R	TTAATTAGCGGCCGCTACAAGCCGGCGGAGACG
ORF60AAF1,<br>annealing for pepORF45(16-40)	GATCCCGTATGCTGCCGATCGAAGGTGCA
ORF60AAF2<br>annealing for pepORF45(16-40)	CCGCGTCGCCGTCCGCCGGTGAAATTTATTTCCCGCCGCCGCCGCTGTC CTCATGC
ORF60AAR1<br>annealing for pepORF45(16-40)	CGGCGGACGGCGACGCGGTGCACCTTCGATCGGCAGCATACGG
ORF60AAR2<br>annealing for pepORF45(16-40)	GGCCGCATGAGGACAGCGGCGGCGGCGGGAAAATAAATTTCAC
ORF45AA16F-BamHI	CAGAGGATCCCGTATGCTGCCGATCGAAGG
ORF45AA76R-NotI	TTAATTAGCGGCCGCTGGTAGCCGGCGGAGACG
Orf-FXFP-MutF	GTCCGCCGGTGAAAGCTGCTGCCGCGCCGCCGCCGC
Orf-FXFP-MutR	GCGGCGGCGGCGCGGCAGCAGCTTTCACCGGCGGAC
ORF-16-SN-XhoI-F	TTAATTCTCGAGCGGTCGTATGCTGCCGATCGAAGG
ORF-76Flag-SN-BglII-R	TTAATTAGATCTTTACTTATCGTCGTCATCCTTGTAATCGCTAGCGGTAGCC GGCGGAGACG
**Cloning and mutagenesis of RSK2**	
RSK2FL-BamHI-Forw	TATAGGATCCATGCCGCTGGCGCAGCTGG
RSK2FL-NheI-F	GATGTTGCTAGCATGCCGCTGGCGCAGC
RSK2FL-Sal-R	TGAATTGTCGACCTACAGGGCTGTTGAGGTGATT
RSK2FL-NotI-Rev	ATAAAATGCGGCCGCTCAGGGCTGTTGAGGTGATTTTTTTAATACC
RSK2N_39-NheI-F	GATGTTGCTAGCAACCCACAAACTGAAGAAGTCAG
RSK2N_351-SalI_R	TGAATTGTCGACCTAAGGCCTGCCCGTTGCAG
RSK2N351-XhoI_Rev	TAATTCTCGAGAGGCCTGCCCGTTGCAG
RSK2N_39-BamHI-F	TTAATTGGATCCAACCCACAAACTGAAGAAGTCAG
RSK2N_351-XhoI-R	TTAATTCTCGAGAGGCCTGCCCGTTGCAG
RSK2FL-SN-XhoI-F	TTTAAACTCGAGCGGTATGCCGCTGGCGCAGC
RSK2FL-SN-NheI-R	AAGTTCGCTAGCTCACAGGGCTGTTGAGGTG
RSK2end-SacI-R	TTTAAAGAGCTCTCACAGGGCTGTTGAGGTG
RSK2FL-SN-NoStop-NheI-R	AAGTTCGCTAGCCAGGGCTGTTGAGGTG
RSK2-L285AF	GATTCTTAAAGCCAAAGCTGGAGCGCCACAGTTTTTGAG
RSK2-L285AR	CTCAAAAACTGTGGCGCTCCAGCTTTGGCTTTAAGAATC

RSK2-F268SF	CTGGTACACTCCCTTCCCAAGGAAAAGATCG
RSK2-F268SR	CGATCTTTTCCTTGGGAAGGGAGTGTACCAG
RSK2-L285WF	CTATGATTCTTAAAGCCAAATGGGGAATGCCACAGTTTTTGAG
RSK2-L285WR	CTCAAAAACTGTGGCATTCCCCATTTGGCTTTAAGAATCATAG
RSK2-M287WF	CTTAAAGCCAAACTTGGATGGCCACAGTTTTTGAGTCC
RSK2-M287WR	GGACTCAAAAACTGTGGCCATCCAAGTTTGGCTTTAAG
RSK2-R305FF	CTTTTACGAATGCTTTTCAAGTTCAATCCTGCAAACAGATTAG
RSK2-R305FR	CTAATCTGTTTGCAGGATTGAACTTGAAAAGCATTCGTAAAAG
RSK2C-414-BamHI-F	TTAATTGGATCCAACAGTATTCAGTTTACTGATGGATATGAAG
RSK2-DeltaDock-XhoI-R	TATATCTCGAGCTGATTACGGTTCAAAGCAGAATATG
**Cloning of VF motif containing peptides and proteins**	
MSK1-23-BamHI-F	AATTAAGGATCCGAGCAGCTCCTCACTGTCAAG
MSK1-348-XhoI-R	TATATACTCGAGAAAGTTACTCACATCTAATTCATCTCG
BMF-BamHI-F	TTAATTGGATCCATGGAGCCATCTCAGTGTGTGGAG
BMF-NotI-R	AATTAAGCGGCCGCTCCTAGGGCCTGCCCC
VFORF-minF,<br>annealing for pepORF45_a	GATCCGATATGAGCGCGCCGGATGATGTGTTTGCGGAAGATACCAGC
VFORF-minR,<br>annealing for pepORF45_a	GGCCGCTGGTATCTTCCGCAAACACATCATCCGGCGCGCTCATATCG
VFORF-midF,<br>annealing for pepORF45_b	GATCCCCGACCGTGATTGATATGAGCGCGCCGGATGATGTGTTTGCGGAA GATACCAGC
VFORF-midR,<br>annealing for pepORF45_b	GGCCGCTGGTATCTTCCGCAAACACATCATCCGGCGCGCTCATATCAATC ACGGTCGGG
VFORF-maxF,<br>annealing for pepORF45_c	GATCCGTGATTGATATGAGCGCGCCGGATGATGTGTTTGCGGAAGATACC CCGAGCCCGCCGGCGACCAGC
VFORF-maxR,<br>annealing for pepORF45_c	GGCCGCTGGTCGCCGGCGGGCTCGGGGTATCTTCCGCAAACACATCATCC GGCGCGCTCATATCAATCACG
SOS1-F1,<br>annealing for pepSOS1	GATCCGATGTGTGCAGCGTGTTTGATAGCGATCATAGCAGCCCGTTTCATA GCAGCAACGATACCGTGTTTATTCAGGTGAC
SOS1-F2,<br>annealing for pepSOS1	GACCCTGCCGCATGGCCCGCGCAGCGCGAGCGTGAGCAGCATTAGCCTG AGC
SOS1-R2,<br>annealing for pepSOS1	GGCCGCTCAGGCTAATGCTGCTCACGCTCGCGCTGCGCGGGCCATGCGG CAGGGTCACCTGAATAAACACGGTATCGT
SOS1-R1,<br>annealing for pepSOS1	TGCTGCTATGAAACGGGCTGCTATGATCGCTATCAAACACGCTGCACACA TCG
YOPM-For,<br>annealing for pepYopM	GATCCGATCCGTATGAATTTGCGCATGAAACCACCGATAAACTGGAAGAT GATGTGTTTGAAAGC
YOPM-Rev,<br>annealing for pepYopM	GGCCGCTTTCAAACACATCATCTTCCAGTTTATCGGTGGTTTCATGCGCA AATTCATACGGATCG
POLG-For,<br>annealing for pepPOLG	GATCCTATCCGGAAGAACTGCTGACCGATGGCGAAGATGATGTGTTTGAT CCGGAACTGGATAGC
POLG-Rev,<br>annealing for pepPOLG	GGCCGCTATCCAGTTCCGGATCAAACACATCATCTTCGCCATCGGTCAGC AGTTCTTCCGGATAG
BMF-For,<br>annealing for pepBMF	GATCCATGGAACCGAGCCAGTGCGTGGAAGAACTGGAAGATGATGTGTT TCAGCCGGAAGATAGC

BMF-Rev,<br>annealing for pepBMF	GGCCGCTATCTTCCGGCTGAAACACATCATCTTCCAGTTCTTCCACGCAC TGGCTCGGTTCCATG
RHDF1-For,<br>annealing for pepRHDF1	GATCCGAAGAACTGAGCACCTATCCGGATGAAGTGTTTGAAAGCCCGAG CGAAGCGGCGCTGAGC
RHDF1-Rev,<br>annealing for pepRHDF1	GGCCGCTCAGCGCCGCTTCGCTCGGGCTTTCAAACACTTCATCCGGATAG GTGCTCAGTTCTTCG
RHDF1-BamHI-F	TATATGGATCCATGAGTGAGGCCCGCAGGGAC
RHDF1-289-XhoI-R	AAATTTCTCGAGCGCTGCCTCCGATGGG
RHDF1-289-VFMut-XhoI-R (F281A)	AAATTTCTCGAGCGCTGCCTCCGATGGGGACTCGGCAACTTCATCCGG
ORF10F10-BamHI-F1,<br>annealing for pepVF-RXXS	GATCCGTGATTGATATGAGCGCGCCGGATGATGTGTTTGCGGAAGATACC CCGAGCCCGCCGGC
ORF10F10-BamHI-F2,<br>annealing for pepVF-RXXS	GACCGGTGGTGGTGGCGGTTCC GGTGGTGGTGGCTCCG
ORF10F10-R2,<br>annealing for pepVF-RXXS	GATCCGGAGCCACCACCACCGGAACCGCCACCACCACCGGTCGCCGGC GGGCTCGGGGTATCTTCC
ORF10F10-R1,<br>annealing for pepVF-RXXS	GCAAACACATCATCCGGCGCGCTCATATCAATCACG
ORF10F10-NotIF,<br>annealing for pepRXXS-VF	GGCCGCGTGATTGATATGAGCGCGCCGGATGATGTGTTTGCGGAAGATAC CCCGAGCCCGCCGGCGACCC
ORF10F10-XhoIR,<br>annealing for pepRXXS-VF	TCGAGGGTCGCCGGCGGGCTCGGGGTATCTTCCGCAAACACATCATCCG GCGCGCTCATATCAATCACGC
**Cloning and mutagenesis of other proteins**	
hPDK1-73-BamHI-F	CAGAGGATCCCAGCCTCGGAAGAAGCGG
hPDK1-358-NotI-R	ATAAATTGCGGCCGCTGAGCTTCGGAGGCGTCTGC
hPDK1-360Stop-NotI-R	AATTAATGCGGCCGCCTAAGCGGTGAGCTTCGGAGGC
DUSP6-NotI-F (MKP3)	AATAATTAGCGGCCGCATGATAGATACGCTCAGACCCGTG
DUSP6-XhoI-R (MKP3)	AATATACTCGAGCGTAGATTGCAGAGAGTCCACC
MEK1F (MKK1)	CGGGATCCATGCCCAAGAAGAAGCCGACGC
MEK1R (MKK1)	ATAGTTTAGCGGCCGCTTAGACGCCAGCAGCATGGGTTC
MEK1SR (MKK1)	ATAGTTTAGCGGCCGCTGACGCCAGCAGCATGGGTTGG
MKK1_4DF	GCAGCTCATCGACGACGATGCCGACGACTTCGTGGGCACAAGG
MKK1_4DR	CCTTGTGCCCACGAAGTCGTCGGCATCGTCGTCGATGAGCTGC
ERK2NheI-F	GACTAGCTAGCATGGCGGCGGCGGCG
ERK2SacI-R	GGACTGAGCTCAGATCTGTATCCTGGCTGG
ERK2-noStop-SacI-R	GGACTGAGCTCAGATCTGTATCCTGGCTGG
p38aNheI-F	CTGAAGGCTAGCATGTCTCAGGAGAGGCC
p38a-noStop-SacI-R	GGAGTCGAGCTCGGACTCCATCTCTTCTTGG
MK2-47-BamHI-F	GATAGGATCCCACGTCAAGTCCGGCCTGCAGATCAAGAAG,
MK2-NotI-R	ATAGTTTAGCGGCCGCTGTGGGCCAGAGCCGCAGC

Supplementary Table 5. Details of SAXS data acquisition, sample details, data analysis and model fitting

**Sample details**	**RSK2**	**RSK2-ERK2**	**RSK2-ERK2-ORF45(16-76)**
Organism	Human	Human	Human/Human herpesvirus 8 type P
Expression Source	BL21 DE3 (E. coli)	BL21 DE3 (E. coli)	BL21 DE3 (E. coli)
Uniprot IDs	P51812	P51812/P28482	P51812/P2848/F5HDE4
Extinction coefficient ε (M^{-1} cm^{-1}, at 280 nm)	79760	124570	126060
Partial specific volume (cm^3 g-1)	0.7425	0.7425	0.7425
Mw from chemical composition (Da)	85003	126677	134383
Concentration ranges (measured at UV 280 nm)	4.8 mg/ml-0.6 mg/ml	6.2 mg/ml-0.4 mg/ml	7.8 mg/ml-0.5 mg/ml
Solvent composition	20mM Tris, pH 8.0, 150mM NaCl,1mM DTT, 1mM TCEP, 10% glycerol	20mM Tris, pH 8.0, 150mM NaCl,1mM DTT, 1mM TCEP, 10% glycerol	20mM Tris, pH 8.0, 150mM NaCl,1mM DTT, 1mM TCEP, 10% glycerol
**SAXS data collection parameters**			
Instrument	P12 at EMBL/DESY PETRA III	P12 at EMBL/DESY PETRA III	P12 at EMBL/DESY PETRA III
Exposure time (s) -number of exposures	0.045x20	0.045x20	0.045x20
Wavelength (Å)	1.24	1.24	1.24
Sample temperature (ºC)	20	20	20
q range (1/nm)	0.028-6.71	0.028-6.71	0.028-6.71
**Software employed**			
Data processing	PRIMUS	PRIMUS	PRIMUS
Data modelling	CORAL	CORAL	CORAL
Molecular graphics	PymMOL	PymMOL	PymMOL
**Structural parameters**			
Guinier Analysis			
$I(0)$	7055±100	12391 ±39	12461±58
R_g (nm)	3.82±0.02	3.94±0.02	3.96±0.02
qRg limits	0.55-1.14	0.56-1.21	0.64-1.23
Fidelity	1	0.97	1
P(r) analysis			
I(0)	6867	12320	12260
R_g (nm)	3.78	3.95	3.91
Dmax (nm)	11.3	12.2	12.5
Volume (VP) (nm^3)	125.25	205.31	224.47
Mw(PR) kDa	86.64	146.09	136.9
Mw(Vc) kDa	83.62	134.2	135.7
**Atomistic modelling**			
CORAL rigid body modelling PDB IDs	3G51,4NIF	3G51,4NIF	7OPO, 4NIF, 7OPM
CORAL presumed flexibility	RSK2 (348-416)	RSK2 (348-416)	RSK2 (348-416), ORF45 (39-50)
CORAL model X^2	0.81	1.07	1.12

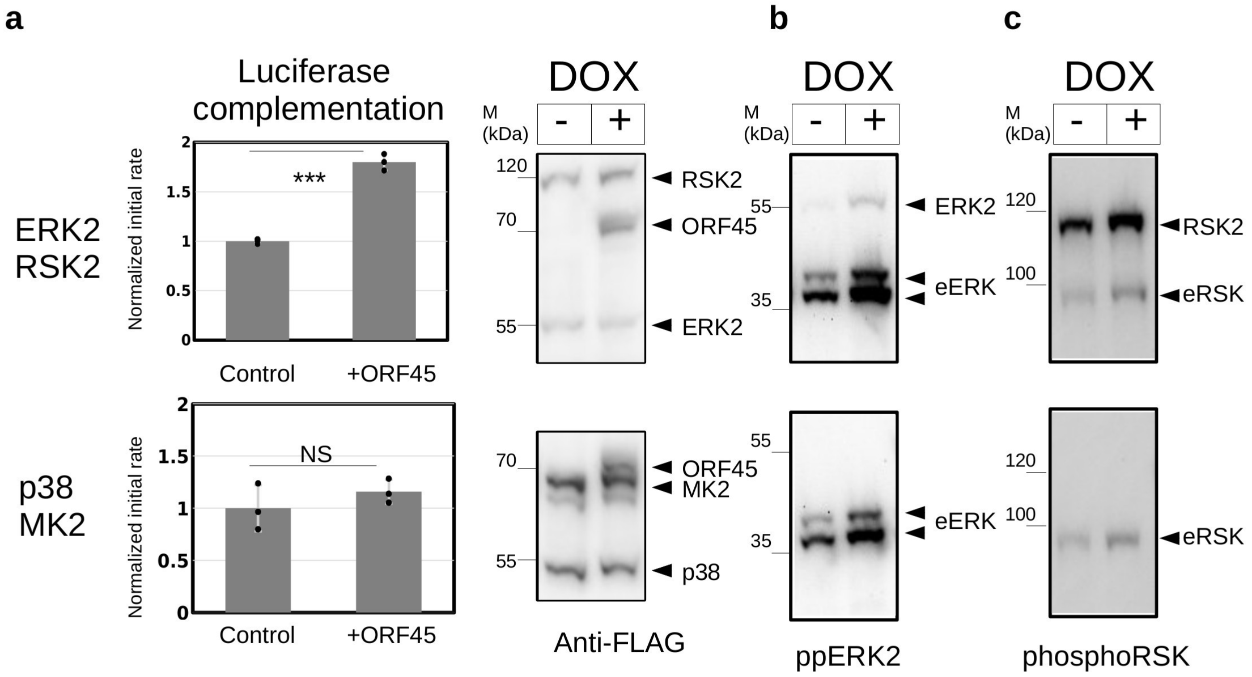

Supplementary Fig. 1: ORF45 specifically promotes ERK-RSK heterodimer formation

(**a**) Protein-protein interactions were probed by using a firefly luciferase complementation assay. ERK2-RSK2 interaction is specifically enhanced compared to p38-MK2 in the presence of stochiometric amounts of ORF45. MAPK, MAPKAPK and ORF45 constructs were expressed with a FLAG-tag and transiently expressed (ERK, RSK, p38 and MK2), or induced by doxycyline (+ORF45). The luminescence signal was normalized to cells which were not treated by doxycycline (Control). Results of the anti-FLAG Western-blots are shown on the right panels. Bar show the mean and error bars indicate SD from three independent experiments. (Paired t-test, two-sided; NS: not significant, *: $p < 0.05$, **: $p < 0.01$, ***: $p < 0.001$).

(**b**, **c**) Phosphorylation of ERK2 (ppERK) and RSK2 (phosphoRSK) is increased in the presence of ORF45. Anti-phospho Western-blots show the phosphorylation of the split luciferase probes – whose luminescence signal was monitored on Panel A, as well as that of endogenous ERK (eERK) or RSK (eRSK). +DOX: after 12 hours of treatment with doxycycline, which induces ORF45 expression (N=1). Source data are provided as Source data file.

a

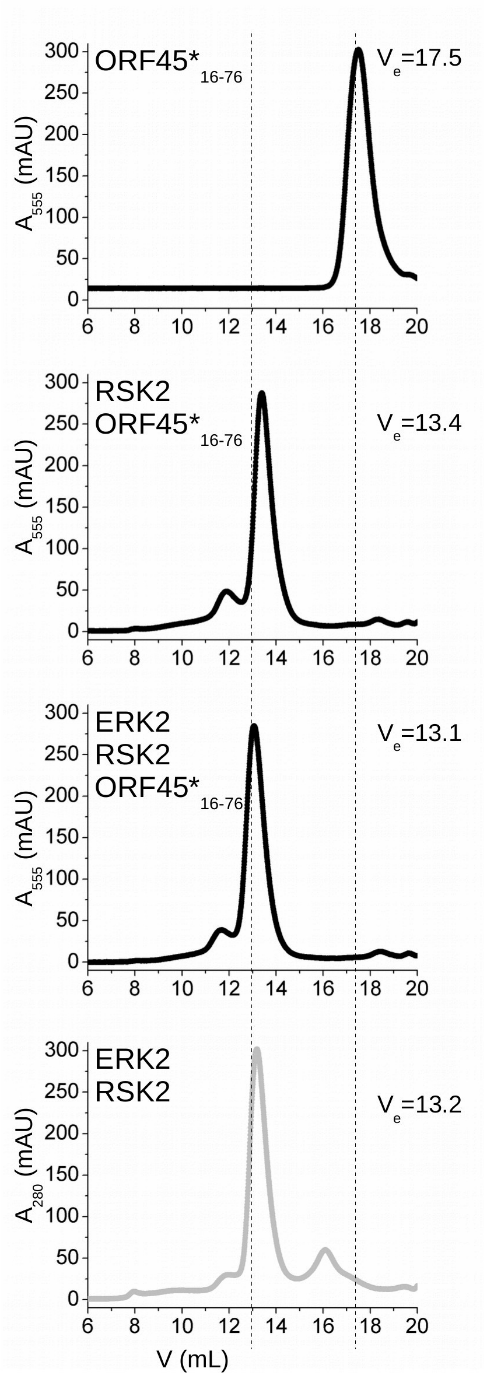

b

	Rg (nm)	Dmax (nm)	Mw(PR) (kDa)	Mw(Vc) (kDa)	Mwt (kDa)	χ^2
RSK2	3.82	11.3±0.2	86.64	83.62	85.00	0.81
RSK2-ERK2	3.94	12.2±0.2	146.09	134.2	126.68	1.07
RSK2-ERK2-ORF45$_{16-76}$	3.96	12.5±0.2	136.9	135.7	134.38	1.47

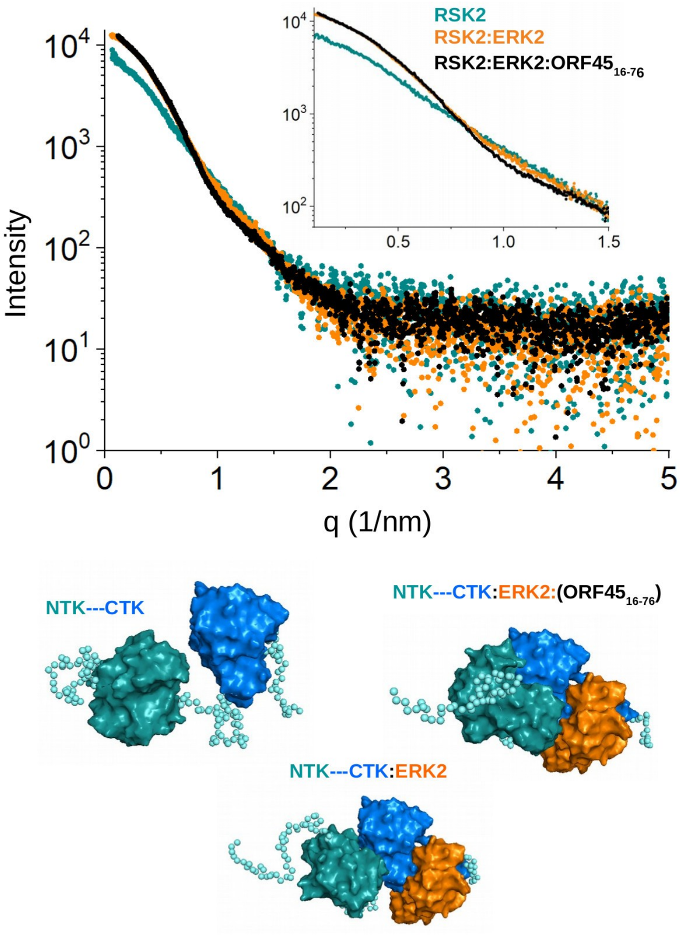

Supplementary Fig. 2: Ternary complex formation analyzed by size exclusion chromatography and SAXS

(**a**) Size exclusion chromatography of different complexes with Alexa555 labeled ORF45(16-76) peptide. V_e: elution volume on a Superdex200 10/30 column. Note that the top three panels show the elution profile measured at 555 nm and monitors the peptide, while the bottom panel shows the elution profile for ERK2-RSK2 monitored at 280 nm.

(**b**) SAXS analysis on RSK2, ERK2-RSK2 and ERK2-RSK2-ORF45(16-76). The table summarizes the radius of gyration (Rg), the maximum diameter (Dmax) and the measured molecular weight of the particles, derived from the Porod volume (Mw(PR)) or from volume correlation (Mw(Vc)). Mwt – calculated molecular weight. χ2 indicates the goodness of the fit to models made by CORAL using crystal structures for the NTK, CTK and the ERK2-CTK complexes[2,3]. Scattering curves of the RSK2, ERK2-RSK2 and ERK2-RSK2-ORF45 samples (with an inset showing the zoomed-in view of the plot) are shown in the middle and the structural models with the best χ2 are shown at the the bottom. Unstructured terminal elements and the interdomain linker are shown with spheres and the kinase domains are shown in surface representation.

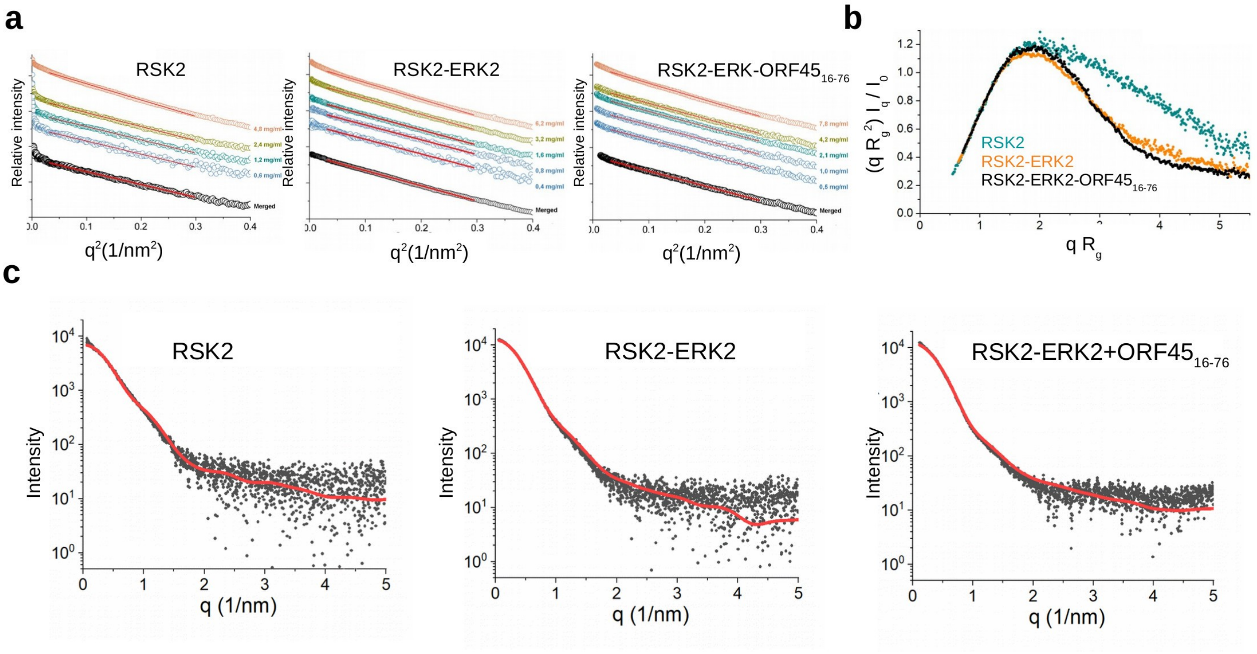

Supplementary Fig. 3: Details of the SAXS analysis

(**a**) Guinier plot analysis of SAXS data. Samples were measured in different concentrations by diluting the stock with the equilibrated buffer obtained when the concentrated stock had been dialyzed. Scattering of each examined complex is shown in the so-called Guinier representation for the whole concentration dilution series (differently colored). The Guinier region used for calculating the radius of gyration (Rg) is highlighted with a red line. The merged curve used for the final Rg calculation is shown in black.

(**b**) Dimensionless Kratky-plots for RSK2, RSK2-ERK2, and RSK2-ERK2-ORF45. Note that compact, globular proteins (complexes) have a bell-shaped (Gaussian) peak, while deviation from this suggests either particle flexibility or asymmetry[4].

(**c**) Modeling of RSK2 and RSK2-ERK2 or RSK2-ERK2+ORF45(16-76) samples based on SAXS data with CORAL. Black dots correspond to experimental data and the red line shows the simulated curve with the best structural model.

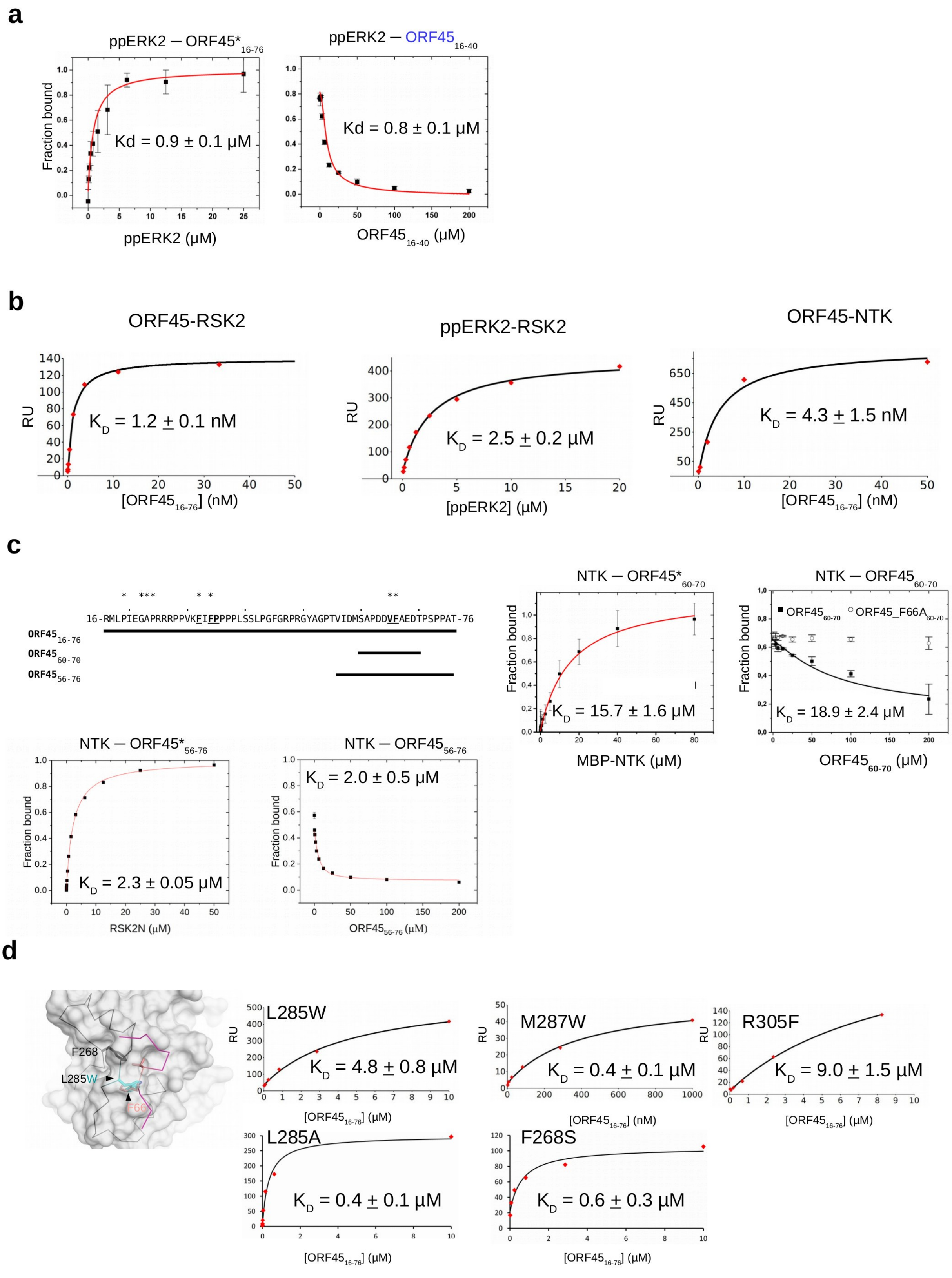

Supplementary Fig. 4: Characterization of ppERK2-ORF45 and ORF45-RSK2 binding

(**a**) Additional data for the ERK-ORF45 binding experiments shown on Fig. 2a. The first panel shows the direct binding curve where the ppERK2 concentration was increased in the presence of

labeled ORF45(16-76), and shows that this longer ORF45 peptide has the same binding affinity compared to ORF45(16-40). The panel on the right shows a competitive binding titration experiment using the unlabeled ORF45(16-40) peptide, and shows that the CF label does not affect binding. Error bars show SD based on three independent experiments. Data points show the mean +/- SD.

(**b**) Equilibrium binding curves of ORF45-RSK2 or ppERK2-RSK2 complexes measured by SPR. Biotin-RSK2 was immobilized on the SPR chip and the analytes (ORF45 or ppERK2) were injected in different concentrations. The NTK and full-length RSK2 bind to the 61 amino acid long ORF45 region with similar affinity (~5 nM or 1 nM, respectively). RU: response units. Note that RU_{max} depends on the amount of RSK2 immobilized on the SPR chip.

(**c**) Binding of ORF45 shorter fragments to RSK2 NTK. Top panels: the left panel shows the fluorescence polarization based direct titration experiment, while the panel on the right compares the binding of the wild-type peptide and its mutated version (F66A) to NTK. (MBP: maltose binding protein fusion tag). Data points indicate the mean and error bars indicate SD from three independent experiments. This data indicates that the short VF motif containing ORF45(60-70) mediates only 20 µM binding to the NTK, in contrast to ORF45(16-76) that binds more than 1000-fold stronger (~5 nM). Lower panels: the panel on the left shows the direct titration experiment with ORF45(56-76) labeled with carboxyfluoresceine, and the panel on right shows the competitive titration experiment with the unlabeled peptide.

(**d**) Validation of the ORF45 binding interface on NTK by mutational analysis. Equilibrium binding curves of ORF45-NTK complexes measured by SPR. Biotin-NTK (mutants: L285W, M287W, R307F, L285A, and F268S) was immobilized on the SPR chip and ORF45(16-76) was injected in different concentrations. RU: response units. Note that RU_{max} depends on the amount of RSK2 immobilized on the SPR chip. The first panel on the left shows the NTK binding slot in surface representation, where the NTK main chain atoms between G264 and L302 - containing the αG and αH - and the ORF45 region between M59 and D69 - containing the VF motif - are shown in gray and magenta, respectively. Note that replacing L285 with tryptophan is incompatible with VF motif binding since F66 from ORF45 would directly clash with the tryptophan side-chain. Moreover, a similar rearrangement as observed in the wild-type protein (L285) upon VF-motif binding is unlikely with the bulkier tryptophan because of F268. Source data are provided as Source data file.

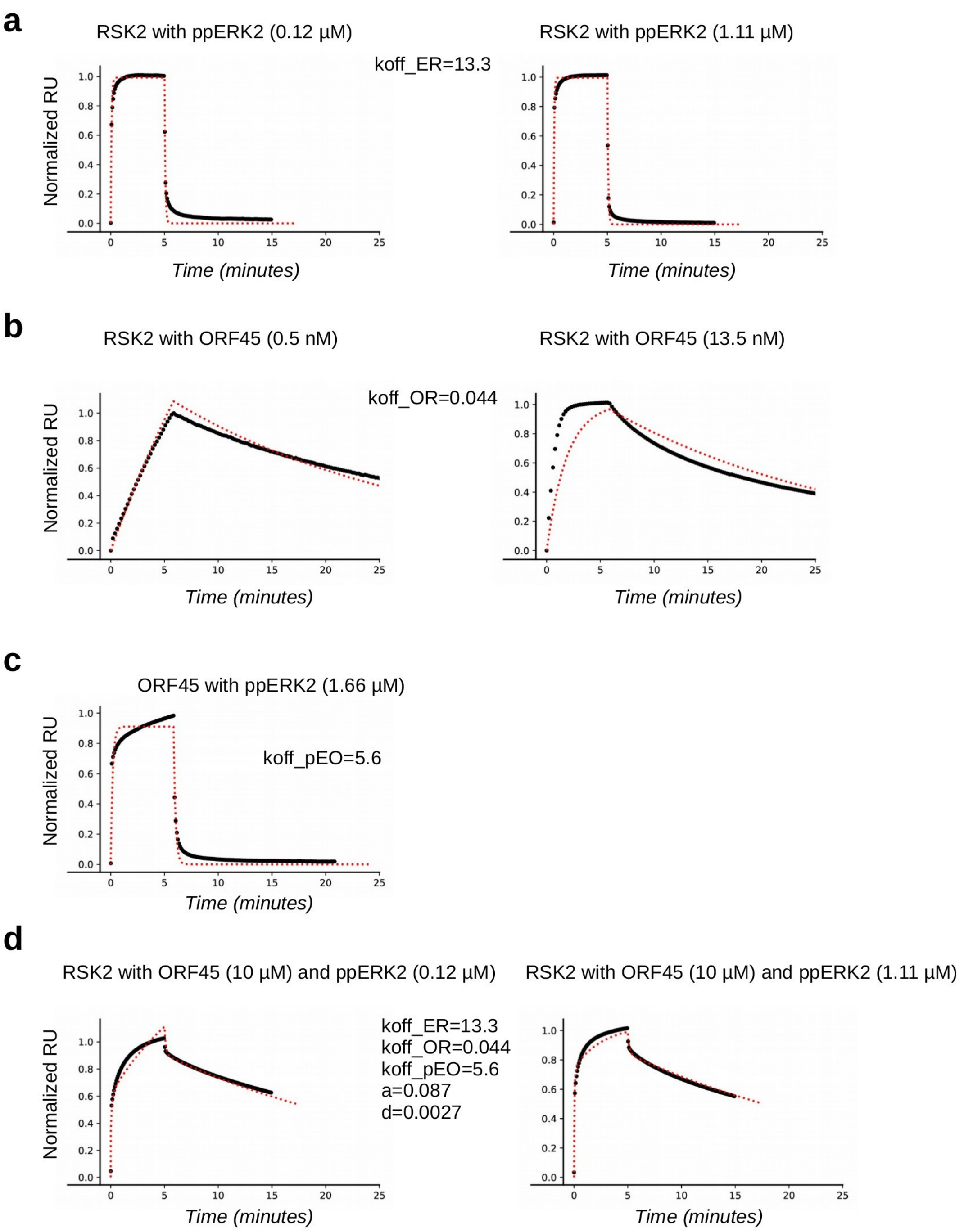

Supplementary Fig. 5: SPR data and simulation for binary and ternary complex assembly

(**a**) ppERK2 binding on the SPR chip with immobilized RSK2. Left and right panels show the experimental data (black) and simulation results (red) with two different concentrations of the analyte injected over the surface (0.12 µM or 1.11 µM ppERK2). These analyte concentrations were chosen to give sensorgrams at about ~ 20 fold less or close to the K_D of the binary complex (see Supplementary Table 3). koff_ER denotes the fitted k_{off} value for ppERK2-RSK2 binding. The SPR signal was normalized to the measured RU_{max} value (see Fig. 4a).

(**b**) ORF45(16-76) binding to the SPR chip with immobilized RSK2. Left and right panels show the experimental data (black) and simulation results (red) with two different concentrations of the analyte injected over the surface (0.5 nM or 13.5 nM ORF45). These analyte concentrations were chosen to give sensorgrams at about ~ 20 fold less or close to the K_D of the binary complex (see Supplementary Table S3). koff_OR denotes the fitted k_{off} value for ORF45-RSK2 binding. The SPR signal was normalized to the measured RU_{max} value (see Fig. 4a).
(**c**) ppERK2 binding to the SPR chip with immobilized ORF45(16-76). koff_pEO denotes the fitted k_{off} value for ppERK2-ORF45 binding.
(**d**) Ternary complex formation on the SPR chip with immobilized RSK2. ORF45(16-76) was mixed together at a fixed high concentration (10 µM) with ppERK2 at two different concentrations (0.12 µM and 1.11 µM). This analyte mix was injected over the RSK2 surface and the experimental data (normalized to the RU_{max} value, see Fig. 4b) was fitted with the model using the equilibrium binding constants (K_D; see Supplementary Table 3) and the corresponding binary koff_ER, koff_OR koff_pEO values from Panel a, b, and c. This fit gave “a” and “d” parameters that affect the k_{on} and the k_{off} of binary binding in the ternary ORF45-ppERK2-RSK2 complex. (RU -response units.)

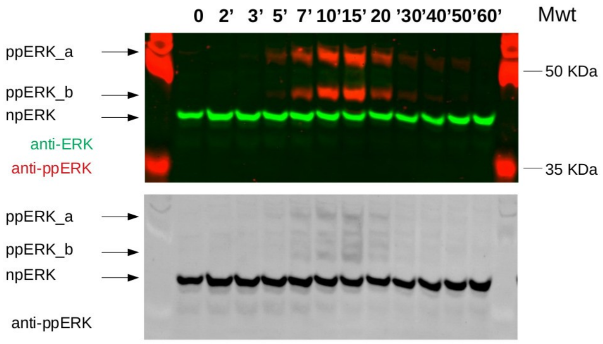

t (min)	pp_a (%)	pp_b (%)	np (%)
5	2	1.5	95.2
7	2.1	2.9	93.6
10	4.8	5.3	87
15	**7.2**	**8.8**	**78.6**
20	4	2.7	89.7

Supplementary Fig. 6: Determination of the ppERK / total ERK ratio upon EGF treatment HEK293 cells were harvested at different type points after EGF treatment. Samples were run on Phospho-tag gels and subjected to Western-blot analysis using a total ERK antibody (anti-ERK in green) and double-phosphorylation specific ERK antibody (anti-ppERK in red in the upper panel) (N=1). The lower panel shows the anti-ERK Western-blot signal only. Western-blot signal ratio for ppERK or total ERK are shown in the table. This analysis identified two distinct bands that were recognized by the anti-ppERK antibody (ppERK_a and ppERK_b) which likely correspond to ppERK1 and ppERK2, or to differently phosphorylated ERK1/2 bands which are both double-phosphorylated (ppERK). (np-nonphosphorylated ERK). Under these conditions, same as in the experiments on Fig. 1a, ~15-20% of the total ERK is double-phosphorylated at 15 minutes.

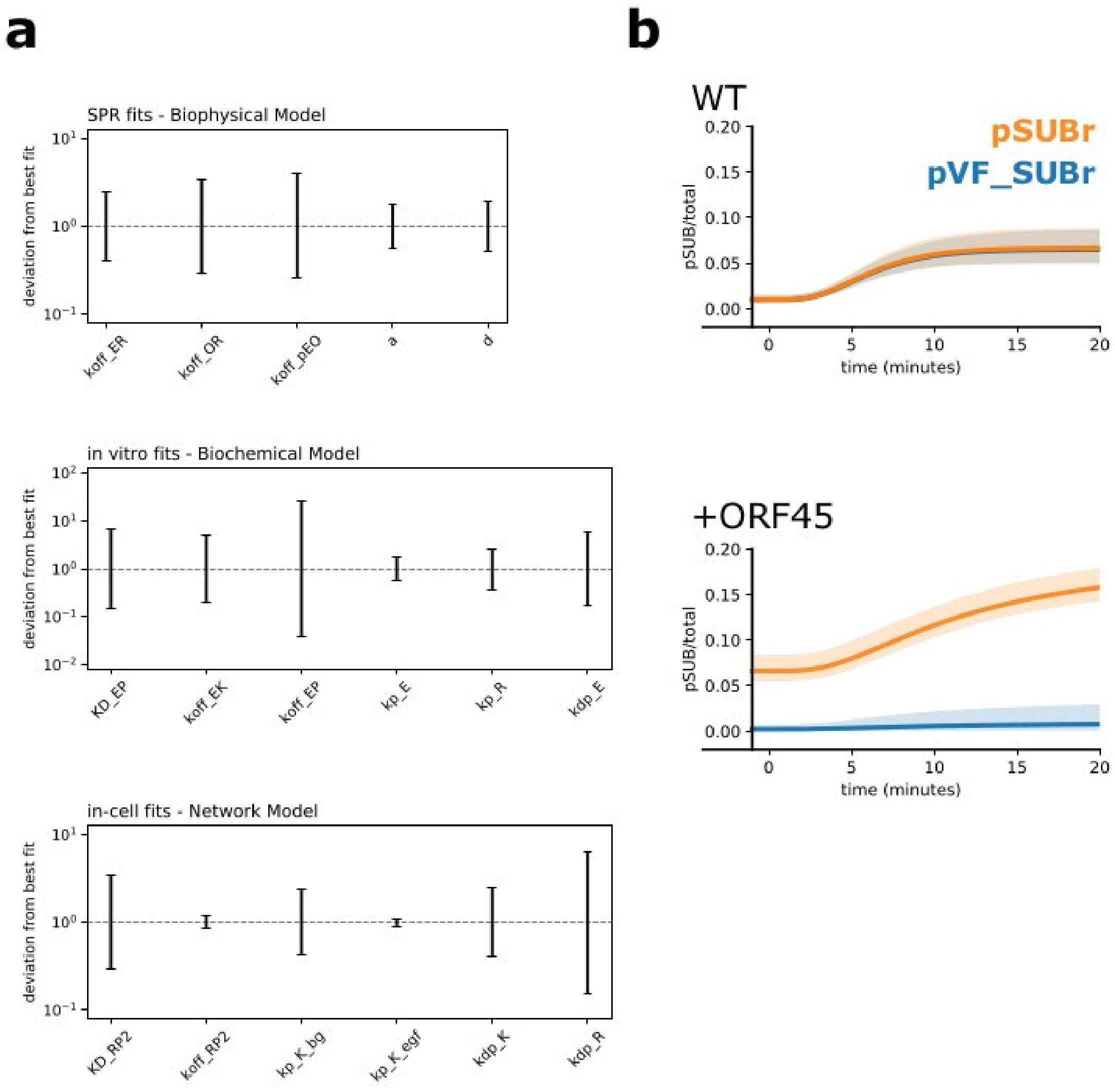

Supplementary Figure 7. Ensemble analysis of model simulations.

(**a**) The spread of parameter values, normalized by the optimal value, is shown for an ensemble of 10.000 parameter sets that are statistically consistent with the experimental data (see Supplementary Methods). Error bars indicate the sample standard deviation and the center is set to 1 and represents the best fit. Note that the spreads of the parameters are within an order of magnitude of the best fit value, indicating that parameters are well constrained by the experimental data.

(**b**) Ensemble prediction corresponding to Fig. 8b. The solid line shows the prediction based on the optimal parameter set. The shaded area represents the full range of the predictions based on all

parameter sets in the ensemble. Note that the different predicted effects of ORF45 on SUBr vs VF-SUBr phosphorylation are maintained when the prediction is based on the whole parameter ensemble.

Supplementary Methods

1 Description of the Model

The model describes the phosphorylation and dephosphorylation of the kinases ERK and RSK and their interaction with the viral protein ORF45. The architecture of the model captures the binding and catalytic events correctly where docking motif, docking groove, and catalytic site accessibility are taken into account based on mechanistic studies. It consists of a set of ordinary differential equations (ODEs) and is simulated and analyzed using the Python package "SloppyCell" and custom written scripts in Python. Two main versions of the model are considered, one that describes the SPR experiments and one that describes the *in vitro* and *in cell* experiments. In addition, there is an extended version of the latter that additionally includes two generic RSK substrate species. All models are available in SBML format as supplementary data.

The model consists of the three "basic" molecular species ERK (called `E` in the model), RSK (`R`), and ORF45 (`O`). Each of these species can bind to the other two individually, but one of the species may also be bound to the two others at the same time, thus giving rise to different variants of ternary complexes. The three species can also form a "closed" complex in which each of the species is bound to both of the others.

Apart from engaging in binding reactions, ERK and RSK can also be phosphorylated and dephosphorylated. In the model the phosphorylated forms are abbreviated as `pE` and `pR`, respectively.

Free ERK is phosphorylated by the activated kinase MKK (`pK` in the model) and dephosphorylated by the phosphatase MKP (`P` in the model). RSK is phosphorylated by phosphorylated ERK. Importantly, RSK can be phosphorylated by ERK only when the latter is not bound to ORF45. RSK is dephosphorylated by a phosphatase that is referred to as `P2` in the model. RSK can only interact with the phosphatase when it is not directly bound to ORF45.

The kinase MKK also occurs in an inactive form (`K`). Its activation is guided by upstream processes that are not represented in detail in the model (see Section 3.3).

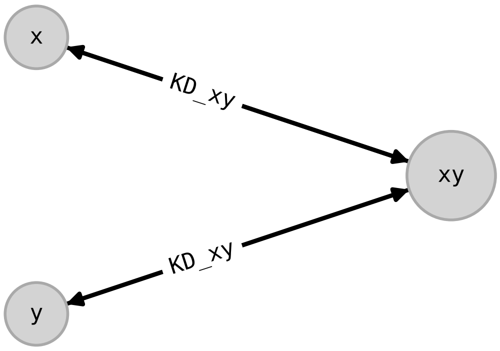

Supplementary Figure 8: Graphical convention.
The reactions occurring in the model are represented using separate arrows from the reactants to the product. Double arrows indicate reversible reactions.

2 Terminology

In order to visualize the model, we use the basic scheme that is illustrated in Supplementary Fig. 8.

All binding reactions and possible complexes are shown in Supplementary Fig. 9. At the center of the diagram are the "basic" species (`O`, `R`, `E`, `pE`, and `pR`). Binary complexes are denoted by combining the letters corresponding to the respective basic species (e.g. `EpR` for the binary complex consisting of

ERK and pRSK). For the ternary complexes the names of the species that directly bind are assembled together to groups, and those groups are then concatenated by underscores. So, for example, pEO_pER refers to the ternary complex in which pERK is directly bound to both ORF45 and RSK, but ORF45 and RSK do not directly bind each other.

Aside from the individual molecular species, we introduce variables that represent the total concentration of each of the molecular species and that are referred to as Otot, Rtot, Etot, pRtot, and pEtot, respectively. They correspond to the sum of concentrations of all species in which the corresponding combination of letters can be found. So, for example, pRtot is the sum of the concentrations of all components that are colored either blue or green in the diagram in Supplementary Fig. 9.

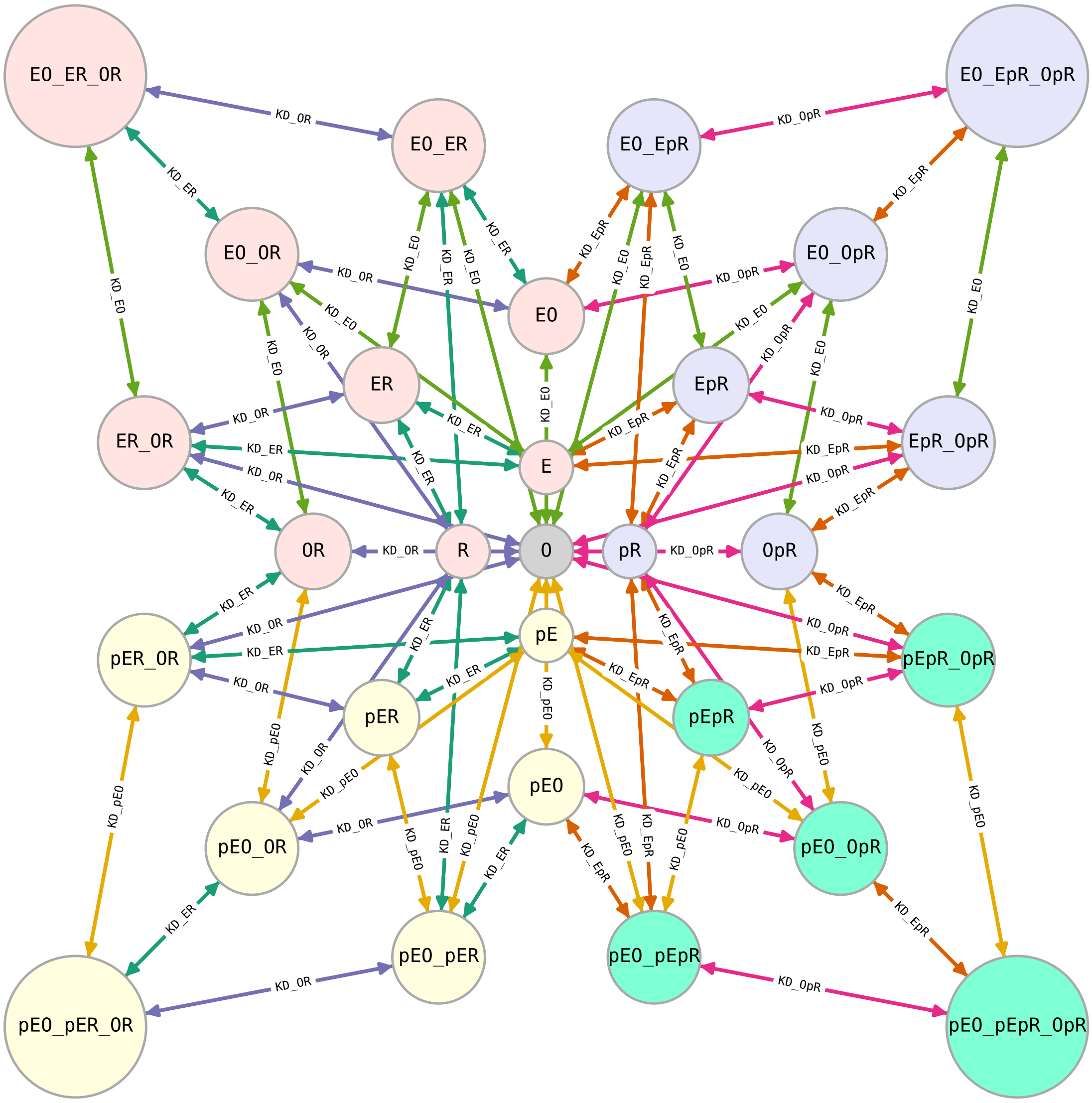

Supplementary Figure 9: Model diagram.
Diagram showing all binding reactions and the possible complexes formed by the three basic components ERK (E), RSK (R), and ORF45 (O). The colors are used to group species that either include the unphosphorylated forms E and R (red), phosphorylated pE and unphosphorylated r (yellow), unphosphorylated E and phosphorylated pR (blue), or both phosphorylated (green). The colors of the arrows serve to highlight reactions with the same dissociation constant.

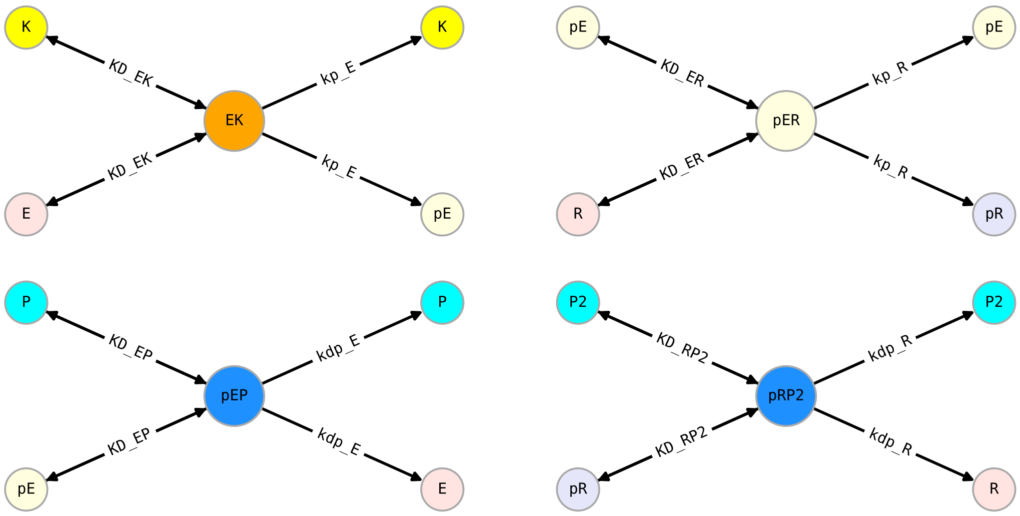

Supplementary Figure 10: Catalytic reactions.
Diagram showing phosphorylation and dephosphorylation in the model. Note that binding reactions are reversible (indicated by double arrows) and catalytic reactions are irreversible. The figure shows only reactions involving the basic species. For the full list of catalytic reactions see section 5.

3 Model Equations

The full model consists of 34 coupled ODEs. Instead of writing them all down explicitly, we provide the general form of the equations, which is based on straightforward mass-action and Michaelis-Menten kinetics. A full list of all reactions that enter into these equations can be found in Section 5.

3.1 Association and Dissociation Reactions

All binding and dissociation reactions are reversible and of the form

$$s_i(\mathtt{x}) + s_j(\mathtt{y}) \underset{\mathtt{koff\_xy}}{\overset{\mathtt{kon\_xy}}{\rightleftharpoons}} s_k(\mathtt{xy}), \tag{1}$$

where $s_i(\mathtt{x})$ and $s_j(\mathtt{y})$ refer to the species $\mathtt{x}$ and $\mathtt{y}$ or to complexes containing these species, and $s_k(\mathtt{xy})$ is a complex in which $\mathtt{x}$ and $\mathtt{y}$ are bound. The parameters $\mathtt{kon\_xy}$ and $\mathtt{koff\_xy}$ are the rates of the forward and reverse reactions, respectively. The corresponding differential equation is of the form

$$\frac{d}{dt}[s_k(\mathtt{xy})] = \mathtt{kon\_xy} \cdot [s_i(\mathtt{x})] \cdot [s_j(\mathtt{y})] - \mathtt{koff\_xy} \cdot [s_k(\mathtt{xy})] + \cdots . \tag{2}$$

The square brackets stand for concentrations, and the dots $(\cdots)$ indicate that there are possible further reactions that contribute to the formation, transformation or dissociation of the complex $s_k(\mathtt{xy})$. The dissociation constant is defined in the usual way as

$$\mathtt{KD\_xy} = \frac{\mathtt{koff\_xy}}{\mathtt{kon\_xy}}. \tag{3}$$

3.2 Catalytic Reactions

Phosphorylation reactions are of the form

$$\mathtt{k} + \mathtt{s} \underset{\mathtt{koff\_ks}}{\overset{\mathtt{kon\_ks}}{\rightleftharpoons}} \mathtt{ks} \xrightarrow{\mathtt{kp\_s}} \mathtt{k} + \mathtt{p}, \tag{4}$$

where k is the kinase, s is the substrate (unphosphorlyated form of a species), and p is the product (the phosphorylated form of the same species). The parameter kp_s is the rate of the irreversible catalytic part of the reaction. Dephosphorylation reactions are of analgous form, using the parameter kdp_s for rate of the catalytic step.

The basic scheme of the catalytic reactions is depicted in Supplementary Fig. 10. Note that kinases and phosphatases bind to their phosphorylated and unphosphorylated substrates with the same rate, but only one of the forms can undergo the catalytic step.

3.3 Activation of the Pathway

For the activation of MKK (the kinase acting upstream of ERK, called K in the model) we use a simplified kinetics of the form

$$\frac{d}{dt}[\mathtt{pK}] = (\mathtt{kp\_K\_bg} + \mathtt{kp\_K\_egf}) \cdot [\mathtt{K}] - \mathtt{kdp\_K} \cdot [\mathtt{pK}], \tag{5}$$

that is, the transition from the the inactive to the active form and its reverse are modeled as single reactions. We distinguish, however, between the activation by the upstream activation of the EGF-pathway (kp_K_egf) and a background activation rate (kp_K_bg) in order to account for the experimental observation that there is a non-zero equilibrium level of pERK already before the stimulation of the pathway.

3.4 The Closed Complex

ERK, RSK, and ORF45 can form a closed ternary complex in which each component is directly bound to the two others. The transition between open ternary complexes and closed complexes has different kinetic properties than the corresponding binary reaction. On the one hand, this is because the conformation of the closed complex may have higher stability leading to a slower dissociation of the binding partners. On the other hand, association occurs between proteins that belong to the same biochemical entity, which makes this reaction different from the usual mass action kinetics. We introduce two parameters, a and d, to account for these differences in the kinetics of association and dissociation, respectively. Supplementary Fig. 11 illustrates this idea schematically.

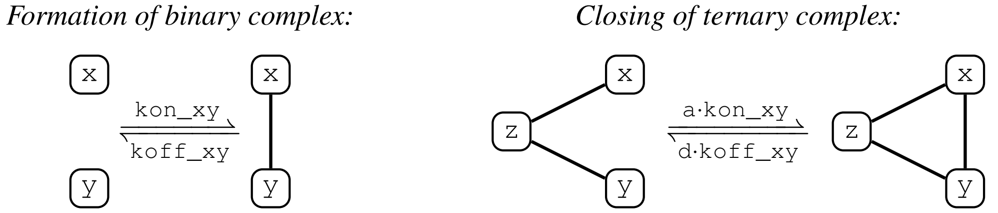

Supplementary Figure 11: Difference in binding kinetics between the formation of a binary complex and the corresponding reaction in the transition from an open to a closed ternary complex. The set of placeholders x, y, and z can be any combination of the basic species (E/pE, R/pR, and O), leading to 8 possibilities of forming a binary and 12 possibilities of closing a ternary complex. While the kon_xy/koff_xy can vary depending on the reaction, the factors a and d are assumed to be the same for all reactions.

The equations that describe the formation of the different closed complexes are of the following form:

$$\frac{d}{dt}[\mathtt{cl}] = \mathtt{a} \cdot \sum_i \mathtt{kon}_i \cdot [\mathtt{op}_i] - \mathtt{d} \cdot \sum_i \mathtt{koff}_i \cdot [\mathtt{cl}]. \tag{6}$$

Here, cl is one of the four closed complexes (corresponding to the four outer vertices in Supplementary Fig. 9). Each of these closed complexes can be formed from three different open precursors that are

referred to as $\texttt{op}_i$. The parameters $\texttt{kon}_i$ and $\texttt{koff}_i$ are the association and dissocation rates of the corresponding binary reactions. The parameter $\texttt{d}$ is a dimensionless number that changes each of the binary dissociation rates by the same factor. The parameter $\texttt{a}$ has a unit of concentration and can be interpreted as the effective concentration that results from bringing the binding partners together in the same complex.

4 Modeling Strategy

We determine plausible ranges for those parameters which were not directly measured by fitting them to different sets of experimental data. We proceed in a step-wise fashion, constraining as many parameters as possible with the simpler SPR and *in vitro* experiments and fitting only the remaining ones to the *in cell* experiments. All parameter values that were used for or obtained from the fitting procedures are listed in Supplementary Table 3.

Because SPR, *in vitro*, and *in cell* experiments are different in many respects, the parameters were not fixed but they were only constrained and were allowed to deviate not much more than by $\pm 10\%$ from the determined value. Note though, that these constraints are not strict, but that deviations add to the overall cost function used for the optimization (For details see the documentation of SloppyCell).

In the main figures we only show the simulations corresponding to the best fit. However, SloppyCell allows for an exhaustive search of the parameter space to generate all parameter sets that are statistically consistent with the data. This is particularly important for so-called "sloppy" models in which many parameters are poorly constrained. As we show, however, the parameters in our model are reasonably well-constrained, and the model predictions still hold even if based on an ensemble fit (Supplementary Fig. 7).

4.1 SPR Experiments

We use the SPR data to obtain the dissociation rates for the binary reactions between ppERK, RSK, and ORF45. In addition, the experiment including all three species allows us to estimate the parameters $\texttt{a}$ and $\texttt{d}$ which describe the behavior of the closed ternary complex.

In SPR experiments a protein of interest (the ligand) is stably bound to a surface and can associate with molecules (the analyte) within a solution that flows along the surface. Therefore, the concentration of the free analyte stays constant in the association phase. Thus, for the analytes in our experiments (ppERK and ORF45), we have the following conservation relations:

$$[\texttt{pE}] = \texttt{pEtot} - \sum_i [s_i(\texttt{pE})] = \text{const.} \tag{7}$$

$$[\texttt{O}] = \texttt{Otot} - \sum_i [s_i(\texttt{O})] = \text{const.}, \tag{8}$$

where, as before, the $s_i(\texttt{E})$, $s_i(\texttt{pE})$ and $s_i(\texttt{o})$ denote all the possible complexes that include $\texttt{E}$ and $\texttt{O}$, respectively.

For the ligands (RSK and ORF45[1]), by contrast, the total amount is constant, leading to the following conservation relation:

$$\texttt{Rtot} = [\texttt{R}] + \sum_i [s_i(\texttt{R})] = \text{const.} \tag{9}$$

$$\texttt{Otot} = [\texttt{O}] + \sum_i [s_i(\texttt{O})] = \text{const.} \tag{10}$$

Another peculiarity of the SPR experiments is that it measures the mass of the complexes and not their concentrations. In order to compare the simulations to the data from the experiments involving two

[1]Note that ORF45 appears both as ligand and as analyte in different experiments.

analytes we therefore need to take into account the relative weight of the different proteins. For this purpose we introduce a new variable

$$\texttt{Rcomp} = \texttt{E\_weight} \cdot \sum_i [s_i(\texttt{ER})] + \texttt{O\_weight} \cdot \sum_i [s_i(\texttt{OR})], \tag{11}$$

where $\texttt{E\_weight}$ and $\texttt{O\_weight}$ denote the relative molecular weights of ERK and ORF45, and the $s_i(\texttt{ER})$ and $s_i(\texttt{OR})$ denote all the complexes with RSK in which ERK and ORF45 occur, respectively. For all our simulations we chose

$$\texttt{O\_weight} = 1 \quad \text{and} \quad \texttt{E\_weight} = 6.3, \tag{12}$$

in agreement with a molecular weight of 6541Da for ORF45 and 41389Da for ERK2. The absolute weights are not important for the modeling because the data are given in arbitrary units, and we allow for a scaling factor that converts model units into the experimental units. This scaling factor is optimized along with the parameter fit. For the same reason, the absolute amount of ligand bound to the surface does not matter for the modeling results. We choose $\texttt{Rtot} = 1\mu M$ and $\texttt{Otot} = 1\mu M$ in the respective experiments.

The model is fit to both the association and the dissociation phase of the experiments. We model the dissociation phase by setting the relevant association rates to zero. All parameters obtained in the fit can be found in Supplementary Table 3.

4.1.1 RSK and ppERK

In this case RSK is the ligand, and ppERK is the analyte. We use a previously measured value of $\texttt{KD\_ER} = 2.5\mu M$ (see Supplementary Fig. 4b and 5a). We fit the model to two experiments with different analyte concentrations of $[\texttt{pE}] = 0.12\mu M$ and $[\texttt{pE}] = 1.11\mu M$.

4.1.2 RSK and ORF45

In this case RSK is the ligand, and ORF45 is the analyte. We use a previously measured value of $\texttt{KD\_OR} = 0.0012\mu M$ (Supplementary Fig. 4b and 5b). We fit the model to two experiments with two different analyte concentrations of $[\texttt{O}] = 0.0005\mu M$ and $[\texttt{O}] = 0.0135\mu M$.

4.1.3 ppERK and ORF45

In this case ORF45 is the ligand, and ppERK is the analyte. We use a previously measured value of $\texttt{KD\_pEO} = 0.8\mu M$ (see Fig. 2a and Supplementary Fig. 5c). We fit the model to one experiment with $[\texttt{pE}] = 1.66\mu M$.

4.1.4 RSK, ppERK, and ORF45

We use the experiment with all three species to obtain values for the parameters $\texttt{a}$ and $\texttt{d}$, which determine the association and dissociation rates for the closed complex. In this case RSK is the ligand, and we have two analytes, ppERK and ORF45 (see Supplementary Fig. 5d). We use the previously measured values of $\texttt{KD\_pEO} = 0.8\mu M$ and $\texttt{KD\_OR} = 0.0012\mu M$, and $\texttt{KD\_ER} = 2.5\mu M$, as well as the values for $\texttt{koff\_ER}$, $\texttt{koff\_OR}$, and $\texttt{koff\_pEO}$ from fitting experiments 4.1.1-4.1.3.

We fit the model to two experiments with two different conentrations for ppERK, $[\texttt{pE}] = 0.12\mu M$ and $[\texttt{pE}] = 1.11\mu M$. The concentration of ORF45 is in both cases $[\texttt{O}] = 10\mu M$.

4.2 *in vitro* Experiments

The *in vitro* experiments are used to obtain estimates for the parameters related to phosphorylation and dephosphorylation and to see whether parameters obtained in the SPR experiments are consistent. We

constrain those parameters that were previously determined by measurements or by fitting the SPR experiments and leave the remaining ones free.

Differently from the SPR experiments, all total species amounts are constant throughout the experiments. Therefore, we have conservation relations of the form

$$\mathtt{xtot} = [\mathtt{x}] + \sum_i [s_i(\mathtt{x})] = \text{const.} \tag{13}$$

for all species x, where xtot denotes the total amount, [x] the concentration of free x, and the $[s_i(\mathtt{x})]$ the concentrations of the different possible complexes in which x appears.

In line with the experimental setup, we allow all binding reactions to have reached an equilibrium when the experimental intervention starts. For this purpose, we run the simulation for $t = 1000\,\text{min}$ before making the parameter changes that correspond to the intervention.

Experiment 1: MKK, ERK, RSK, ± ORF45 In this experiment MKK, (unphosphorylated) ERK, and RSK are mixed together with or without ORF45 (Fig. 5a). The initiation of kinase activity is modeled by setting the parameters kcatkin and kcatrp, which determine the phosphorylation of ERK and RSK, respectively, from zero to a positive value at $t = 1000\,\text{min}$. In line with the experiment, the initial concentrations are:

species	value (μM)
Etot	2.0
pE	0.0
Ktot	0.25
Rtot	2.0
Otot	0.0 or 50.0

Experiment 2: ppERK, MKP, ±RSK, ±ORF45 In this experiment (phosphorylated) ppERK is mixed with MKP in the presence or absence of RSK and ORF45 (Fig. 5c). The initiation of phosphatase activity is modeled by setting the parameter Ptot, corresponding to the total amount of MKP, from zero to $1\mu M$ at $t = 1000\,\text{min}$. In line with the experiment, the initial conditions are:

species	value (μM)
Etot	1.0
pE	1.0
Ptot	$0 \rightarrow 1$
Rtot	0.0 or 1.0
Otot	0.0 or 5.0

The optimal parameters resulting from fitting the *in vitro* experiments can be found in Supplementary Table 3. Fig. 6b of the main text shows a visual comparison of data and model simulations.

4.3 *in cell* Experiments

We assume that the *in cell* and the *in vitro* experiments can be modeled in a similar way. That is, we assume that all species are well-mixed and conserved. Additionally, we now also introduce a phosphatase acting on RSK (called P2), and we include the activation reaction of MKK (K) as described in Equation (5). As in the *in vitro* experiments, we allow the system to find an equilibrium, but then the stimulus is simulated by setting the parameter kp_K_egf from zero to a positive value. The initial concentrations are

species	value (μM)
Ktot	1.2
K	1.2
Etot	0.7
Rtot	2.0
Otot	1.0
Ptot	1.0
P2tot	1.0

The optimal parameters resulting from fitting the *in cell* experiments can be found in Supplementary Table 3. Fig. 6c of the main text shows a visual comparison of data and model simulations.

4.4 Model Predictions and Model with Substrates

The model predictions were obtained by simulating the model in the same way as in 4.3, using the optimal *in cell* parameter set, except for the indicated changes (see Fig. 6d).

To simulate the effects of different RSK substrates competing with ORF45 (Fig. 8b), we added two species `SUBC` and `SUBF` to the model. `SUBC` competes with ORF45, meaning that it can only bind to `pR` if `pR` is not directly bound to `O`. `SUBF`, by contrast, can interact with all complexes containing `pR`. The interactions of both substrates are guided by the parameters `kon_pRSUB`, `koff_pRSUB`, `kp_pRSUB`, and `kdp_pRSUB`, which we all set to 1 in our simulations since here we are only interested in qualitative effects. We set the total amounts of the two substrates to a small value (0.01) in order to ensure that the behavior of the rest of the model is not affected (i.e. the substrates are only used as read-outs).

5 Full List of Model Reactions

ERK RSK binding

Reaction	Rate law
E + R ⇋ ER	kon_ER · [E] · [R] − koff_ER · [ER]
pE + R ⇋ pER	kon_ER · [pE] · [R] − koff_ER · [pER]
EO + R ⇋ EO_ER	kon_ER · [EO] · [R] − koff_ER · [EO_ER]
E + OR ⇋ ER_OR	kon_ER · [E] · [OR] − koff_ER · [ER_OR]
pEO + R ⇋ pEO_pER	kon_ER · [pEO] · [R] − koff_ER · [pEO_pER]
pE + OR ⇋ pER_OR	kon_ER · [pE] · [OR] − koff_ER · [pER_OR]
EO_OR ⇋ EO_ER_OR	a · kon_ER · [EO_OR] − d · koff_ER · [EO_ER_OR]
pEO_OR ⇋ pEO_pER_OR	a · kon_ER · [pEO_OR] − d · koff_ER · [pEO_pER_OR]
E + RP2 ⇋ ERP2	kon_ER · [E] · [RP2] − koff_ER · [ERP2]
pE + RP2 ⇋ pERP2	kon_ER · [pE] · [RP2] − koff_ER · [pERP2]
EO + RP2 ⇋ EO_ERP2	kon_ER · [EO] · [RP2] − koff_ER · [EO_ERP2]
pEO + RP2 ⇋ pEO_pERP2	kon_ER · [pEO] · [RP2] − koff_ER · [pEO_pERP2]

ERK ORF binding

Reaction	Rate law
E + O ⇋ EO	kon_EO · [E] · [O] − koff_EO · [EO]
E + OR ⇋ EO_OR	kon_EO · [E] · [OR] − koff_EO · [EO_OR]
ER + O ⇋ EO_ER	kon_EO · [ER] · [O] − koff_EO · [EO_ER]
E + OpR ⇋ EO_OpR	kon_EO · [E] · [OpR] − koff_EO · [EO_OpR]
EpR + O ⇋ EO_EpR	kon_EO · [EpR] · [O] − koff_EO · [EO_EpR]
ER_OR ⇋ EO_ER_OR	a · kon_EO · [ER_OR] − d · koff_EO · [EO_ER_OR]
EpR_OpR ⇋ EO_EpR_OpR	a · kon_EO · [EpR_OpR] − d · koff_EO · [EO_EpR_OpR]
ERP2 + O ⇋ EO_ERP2	kon_EO · [ERP2] · [O] − koff_EO · [EO_ERP2]
EpRP2 + O ⇋ EO_EpRP2	kon_EO · [EpRP2] · [O] − koff_EO · [EO_EpRP2]
EP + O ⇋ EPO	kon_EO · [EP] · [O] − koff_EO · [EPO]
EP + OR ⇋ EPO_OR	kon_EO · [EP] · [OR] − koff_EO · [EPO_OR]
EP + OpR ⇋ EPO_OpR	kon_EO · [EP] · [OpR] − koff_EO · [EPO_OpR]
EpK + O ⇋ EpKO	kon_EO · [EpK] · [O] − koff_EO · [EpKO]
EpK + OR ⇋ EpKO_OR	kon_EO · [EpK] · [OR] − koff_EO · [EpKO_OR]
EpK + OpR ⇋ EpKO_OpR	kon_EO · [EpK] · [OpR] − koff_EO · [EpKO_OpR]
EK + O ⇋ EKO	kon_EO · [EK] · [O] − koff_EO · [EKO]
EK + OR ⇋ EKO_OR	kon_EO · [EK] · [OR] − koff_EO · [EKO_OR]
EK + OpR ⇋ EKO_OpR	kon_EO · [EK] · [OpR] − koff_EO · [EKO_OpR]

ppERK ORF binding

Reaction	Rate law
pE + O ⇋ pEO	kon_EO · [pE] · [O] − koff_pEO · [pEO]
pE + OR ⇋ pEO_OR	kon_EO · [pE] · [OR] − koff_pEO · [pEO_OR]
pER + O ⇋ pEO_pER	kon_EO · [pER] · [O] − koff_pEO · [pEO_pER]
pE + OpR ⇋ pEO_OpR	kon_EO · [pE] · [OpR] − koff_pEO · [pEO_OpR]
pEpR + O ⇋ pEO_pEpR	kon_EO · [pEpR] · [O] − koff_pEO · [pEO_pEpR]
pER_OR ⇋ pEO_pER_OR	a · kon_EO · [pER_OR] − d · koff_pEO · [pEO_pER_OR]
pEpR_OpR ⇋ pEO_pEpR_OpR	a · kon_EO · [pEpR_OpR] − d · koff_pEO · [pEO_pEpR_OpR]
pERP2 + O ⇋ pEO_pERP2	kon_EO · [pERP2] · [O] − koff_pEO · [pEO_pERP2]
pEpRP2 + O ⇋ pEO_pEpRP2	kon_EO · [pEpRP2] · [O] − koff_pEO · [pEO_pEpRP2]
pEP + O ⇋ pEPO	kon_EO · [pEP] · [O] − koff_pEO · [pEPO]
pEP + OR ⇋ pEPO_OR	kon_EO · [pEP] · [OR] − koff_pEO · [pEPO_OR]
pEP + OpR ⇋ pEPO_OpR	kon_EO · [pEP] · [OpR] − koff_pEO · [pEPO_OpR]
pEpK + O ⇋ pEpKO	kon_EO · [pEpK] · [O] − koff_pEO · [pEpKO]
pEpK + OR ⇋ pEpKO_OR	kon_EO · [pEpK] · [OR] − koff_pEO · [pEpKO_OR]
pEpK + OpR ⇋ pEpKO_OpR	kon_EO · [pEpK] · [OpR] − koff_pEO · [pEpKO_OpR]
pEK + O ⇋ pEKO	kon_EO · [pEK] · [O] − koff_pEO · [pEKO]
pEK + OR ⇋ pEKO_OR	kon_EO · [pEK] · [OR] − koff_pEO · [pEKO_OR]
pEK + OpR ⇋ pEKO_OpR	kon_EO · [pEK] · [OpR] − koff_pEO · [pEKO_OpR]

ORF RSK binding

Reaction	Rate law
O + R ⇋ OR	kon_OR · [O] · [R] − koff_OR · [OR]
EO + R ⇋ EO_OR	kon_OR · [EO] · [R] − koff_OR · [EO_OR]
O + ER ⇋ ER_OR	kon_OR · [O] · [ER] − koff_OR · [ER_OR]
pEO + R ⇋ pEO_OR	kon_OR · [pEO] · [R] − koff_OR · [pEO_OR]
O + pER ⇋ pER_OR	kon_OR · [O] · [pER] − koff_OR · [pER_OR]
EO_ER ⇋ EO_ER_OR	a · kon_OR · [EO_ER] − d · koff_OR · [EO_ER_OR]
pEO_pER ⇋ pEO_pER_OR	a · kon_OR · [pEO_pER] − d · koff_OR · [pEO_pER_OR]
EPO + R ⇋ EPO_OR	kon_OR · [EPO] · [R] − koff_OR · [EPO_OR]
pEPO + R ⇋ pEPO_OR	kon_OR · [pEPO] · [R] − koff_OR · [pEPO_OR]
EpKO + R ⇋ EpKO_OR	kon_OR · [EpKO] · [R] − koff_OR · [EpKO_OR]
pEpKO + R ⇋ pEpKO_OR	kon_OR · [pEpKO] · [R] − koff_OR · [pEpKO_OR]
EKO + R ⇋ EKO_OR	kon_OR · [EKO] · [R] − koff_OR · [EKO_OR]
pEKO + R ⇋ pEKO_OR	kon_OR · [pEKO] · [R] − koff_OR · [pEKO_OR]

ORF pRSK binding

Reaction	Rate law
O + pR ⇋ OpR	kon_OR · [O] · [pR] − koff_OpR · [OpR]
EO + pR ⇋ EO_OpR	kon_OR · [EO] · [pR] − koff_OpR · [EO_OpR]
O + EpR ⇋ EpR_OpR	kon_OR · [O] · [EpR] − koff_OpR · [EpR_OpR]
pEO + pR ⇋ pEO_OpR	kon_OR · [pEO] · [pR] − koff_OpR · [pEO_OpR]
O + pEpR ⇋ pEpR_OpR	kon_OR · [O] · [pEpR] − koff_OpR · [pEpR_OpR]
EO_EpR ⇋ EO_EpR_OpR	a · kon_OR · [EO_EpR] − d · koff_OpR · [EO_EpR_OpR]
pEO_pEpR ⇋ pEO_pEpR_OpR	a · kon_OR · [pEO_pEpR] − d · koff_OpR · [pEO_pEpR_OpR]
EPO + pR ⇋ EPO_OpR	kon_OR · [EPO] · [pR] − koff_OpR · [EPO_OpR]
pEPO + pR ⇋ pEPO_OpR	kon_OR · [pEPO] · [pR] − koff_OpR · [pEPO_OpR]
EpKO + pR ⇋ EpKO_OpR	kon_OR · [EpKO] · [pR] − koff_OpR · [EpKO_OpR]
pEpKO + pR ⇋ pEpKO_OpR	kon_OR · [pEpKO] · [pR] − koff_OpR · [pEpKO_OpR]
EKO + pR ⇋ EKO_OpR	kon_OR · [EKO] · [pR] − koff_OpR · [EKO_OpR]
pEKO + pR ⇋ pEKO_OpR	kon_OR · [pEKO] · [pR] − koff_OpR · [pEKO_OpR]

ERK MKK binding

Reaction	Rate law
E + K ⇋ EK	kon_EK · [E] · [K] − koff_EK · [EK]
pE + K ⇋ pEK	kon_EK · [pE] · [K] − koff_EK · [pEK]
E + pK ⇋ EpK	kon_EK · [E] · [pK] − koff_EK · [EpK]
pE + pK ⇋ pEpK	kon_EK · [pE] · [pK] − koff_EK · [pEpK]
EO + pK ⇋ EpKO	kon_EK · [EO] · [pK] − koff_EK · [EpKO]
pEO + pK ⇋ pEpKO	kon_EK · [pEO] · [pK] − koff_EK · [pEpKO]
EO_OR + pK ⇋ EpKO_OR	kon_EK · [EO_OR] · [pK] − koff_EK · [EpKO_OR]
pEO_OR + pK ⇋ pEpKO_OR	kon_EK · [pEO_OR] · [pK] − koff_EK · [pEpKO_OR]
EO_OpR + pK ⇋ EpKO_OpR	kon_EK · [EO_OpR] · [pK] − koff_EK · [EpKO_OpR]
pEO_OpR + pK ⇋ pEpKO_OpR	kon_EK · [pEO_OpR] · [pK] − koff_EK · [pEpKO_OpR]
EO + K ⇋ EKO	kon_EK · [EO] · [K] − koff_EK · [EKO]
pEO + K ⇋ pEKO	kon_EK · [pEO] · [K] − koff_EK · [pEKO]
EO_OR + K ⇋ EKO_OR	kon_EK · [EO_OR] · [K] − koff_EK · [EKO_OR]
pEO_OR + K ⇋ pEKO_OR	kon_EK · [pEO_OR] · [K] − koff_EK · [pEKO_OR]
EO_OpR + K ⇋ EKO_OpR	kon_EK · [EO_OpR] · [K] − koff_EK · [EKO_OpR]
pEO_OpR + K ⇋ pEKO_OpR	kon_EK · [pEO_OpR] · [K] − koff_EK · [pEKO_OpR]

ERK MKP binding

Reaction	Rate law
E + P ⇋ EP	kon_EP · [E] · [P] − koff_EP · [EP]
pE + P ⇋ pEP	kon_EP · [pE] · [P] − koff_EP · [pEP]
EO + P ⇋ EPO	kon_EP · [EO] · [P] − koff_EP · [EPO]
pEO + P ⇋ pEPO	kon_EP · [pEO] · [P] − koff_EP · [pEPO]
EO_OR + P ⇋ EPO_OR	kon_EP · [EO_OR] · [P] − koff_EP · [EPO_OR]
pEO_OR + P ⇋ pEPO_OR	kon_EP · [pEO_OR] · [P] − koff_EP · [pEPO_OR]
EO_OpR + P ⇋ EPO_OpR	kon_EP · [EO_OpR] · [P] − koff_EP · [EPO_OpR]
pEO_OpR + P ⇋ pEPO_OpR	kon_EP · [pEO_OpR] · [P] − koff_EP · [pEPO_OpR]

RSK PP2 binding

Reaction	Rate law
pR + P2 ⇋ pRP2	kon_RP2 · [pR] · [P2] − koff_RP2 · [pRP2]
R + P2 ⇋ RP2	kon_RP2 · [R] · [P2] − koff_RP2 · [RP2]
ER + P2 ⇋ ERP2	kon_RP2 · [ER] · [P2] − koff_RP2 · [ERP2]
pER + P2 ⇋ pERP2	kon_RP2 · [pER] · [P2] − koff_RP2 · [pERP2]
EO_ER + P2 ⇋ EO_ERP2	kon_RP2 · [EO_ER] · [P2] − koff_RP2 · [EO_ERP2]
pEO_pER + P2 ⇋ pEO_pERP2	kon_RP2 · [pEO_pER] · [P2] − koff_RP2 · [pEO_pERP2]
EpR + P2 ⇋ EpRP2	kon_RP2 · [EpR] · [P2] − koff_RP2 · [EpRP2]
pEpR + P2 ⇋ pEpRP2	kon_RP2 · [pEpR] · [P2] − koff_RP2 · [pEpRP2]
EO_EpR + P2 ⇋ EO_EpRP2	kon_RP2 · [EO_EpR] · [P2] − koff_RP2 · [EO_EpRP2]
pEO_pEpR + P2 ⇋ pEO_pEpRP2	kon_RP2 · [pEO_pEpR] · [P2] − koff_RP2 · [pEO_pEpRP2]

ERK phosphorylation/dephosphorylation

Reaction	Rate law
EpK → pE + pK	kp_E · [EpK]
pEP → E + P	kdp_E · [pEP]

RSK phosphorylation/dephosphorylation

Reaction	Rate law
pER_OR → OpR + pE	kp_R · [pER_OR]
pER → pR + pE	kp_R · [pER]
pRP2 → R + P2	kdp_R · [pRP2]
EpRP2 → ER + P2	kdp_R · [EpRP2]
pEpRP2 → pER + P2	kdp_R · [pEpRP2]
EO_EpRP2 → EO_ER + P2	kdp_R · [EO_EpRP2]
pEO_pEpRP2 → pEO_ER + P2	kdp_R · [pEO_pEpRP2]

MAPK activation

Reaction	Rate law
K ⇋ pK	(kp_K_bg + kp_K_egf) · [K] − kdp_K · [pK]

Supplementary References

[1] Gògl, G. *et al.* Dynamic control of RSK complexes by phosphoswitch-based regulation. *FEBS J.* **285**, 46–71 (208).

[2] Ikuta, M. *et al.* Crystal structures of the N-terminal kinase domain of human RSK1 bound to three different ligands: Implications for the design of RSK1 specific inhibitors. *Protein Sci.* **16**, 2626–2635 (2007).

[3] Alexa, A. *et al.* Structural assembly of the signaling competent ERK2-RSK1 heterodimeric protein kinase complex. *Proc. Natl. Acad. Sci. U. S. A.* **112**, 2711–2716 (2015).

[4] Durand, D. *et al.* NADPH oxidase activator p67phox behaves in solution as a multidomain protein with semi-flexible linkers. *J. Struct. Biol.* **169**, 45–53 (2010).
