# Supplementary material for: A non-catalytic herpesviral protein reconfigures ERK-RSK signaling by targeting kinase docking systems in the host
Source: Nat Commun. 2022 Jan 25;13:472. doi: 10.1038/s41467-022-28109-x (PMC8789800; doi:10.1038/s41467-022-28109-x)
Supplement: Supplementary file 3 — Description of Additianal supplementary files [file 41467_2022_28109_MOESM3_ESM.doc]

File Name: Supplementary Data 1

Description: The zip file contains three .xml files in SBML format for the SPR and the in vitro/in-cell models used in this study with a README.txt file.
