# Supplementary material for: A non-catalytic herpesviral protein reconfigures ERK-RSK signaling by targeting kinase docking systems in the host
Source: Nat Commun. 2022 Jan 25;13:472. doi: 10.1038/s41467-022-28109-x (PMC8789800; doi:10.1038/s41467-022-28109-x)
Supplement: Supplementary file 5 — Reporting summary [file 41467_2022_28109_MOESM5_ESM.pdf]

nature portfolio

Corresponding author(s): Attila Remenyi

Last updated by author(s): 2021/12/20

Reporting Summary

Nature Portfolio wishes to improve the reproducibility of the work that we publish. This form provides structure for consistency and transparency in reporting. For further information on Nature Portfolio policies, see our Editorial Policies and the Editorial Policy Checklist.

Statistics

For all statistical analyses, confirm that the following items are present in the figure legend, table legend, main text, or Methods section.

n/a	Confirmed	
☐	☒	The exact sample size (*n*) for each experimental group/condition, given as a discrete number and unit of measurement
☐	☒	A statement on whether measurements were taken from distinct samples or whether the same sample was measured repeatedly
☐	☒	The statistical test(s) used AND whether they are one- or two-sided<br>*Only common tests should be described solely by name; describe more complex techniques in the Methods section.*
☒	☐	A description of all covariates tested
☒	☐	A description of any assumptions or corrections, such as tests of normality and adjustment for multiple comparisons
☐	☒	A full description of the statistical parameters including central tendency (e.g. means) or other basic estimates (e.g. regression coefficient) AND variation (e.g. standard deviation) or associated estimates of uncertainty (e.g. confidence intervals)
☐	☒	For null hypothesis testing, the test statistic (e.g. *F*, *t*, *r*) with confidence intervals, effect sizes, degrees of freedom and *P* value noted<br>*Give P values as exact values whenever suitable.*
☒	☐	For Bayesian analysis, information on the choice of priors and Markov chain Monte Carlo settings
☒	☐	For hierarchical and complex designs, identification of the appropriate level for tests and full reporting of outcomes
☒	☐	Estimates of effect sizes (e.g. Cohen's *d*, Pearson's *r*), indicating how they were calculated

Our web collection on statistics for biologists contains articles on many of the points above.

Software and code

Policy information about availability of computer code

Data collection	P12 beam line EMBL-Hamburg (PETRA) and BM29 beamline at ESRF
Data analysis	Crystallography: XDS (BUILT=20190315), CCP4i 7.0.0.78, AIMLESS 0.7.4, COOT 0.9, PHENIX 1.17.1-3660, Pymol 1.8<br>SAXS: ATSAS 3.0, PRIMUS 3.1, CORAL 1.1<br>HDX-MS: PLG 2.5, DynamX 3.0, DECA 1.14<br>SPR: Biacore S200 Evaluation Software 1.1<br>Srtuctural modeling: MODELLER 10.1<br>Signal network modeling: SlopyCell 1.1.0<br>Quantitative western blot analysis: Image Studio Lite 5.2<br>Data fitting and visualization: OriginPro 8, Qtiplot 0.9.8.9<br>Statistical analysis and visualization: LibreOffice Calc 6.0.7.3

For manuscripts utilizing custom algorithms or software that are central to the research but not yet described in published literature, software must be made available to editors and reviewers. We strongly encourage code deposition in a community repository (e.g. GitHub). See the Nature Portfolio guidelines for submitting code & software for further information.

Data

Policy information about availability of data

All manuscripts must include a data availability statement. This statement should provide the following information, where applicable:

- Accession codes, unique identifiers, or web links for publicly available datasets
- A description of any restrictions on data availability
- For clinical datasets or third party data, please ensure that the statement adheres to our policy

The HDX-MS data was deposited in a ProteomeXchange repository with the accession code: PXD030612. The crystal structure of the ppERK2-ORF45(27-40) and the RSK2 NTK-ORF45 complexes were deposited in the Protein Data Bank with accession codes 7OPM and 7OPO, respectively. The models and all code to reproduce the simulations contained in this study can be found here. The following X-ray structures are available from the PDB: 4NIF, 2ERK, 3G51, and 4NW6. Source data are provided with this paper.

Field-specific reporting

Please select the one below that is the best fit for your research. If you are not sure, read the appropriate sections before making your selection.

[x] Life sciences [ ] Behavioural & social sciences [ ] Ecological, evolutionary & environmental sciences

For a reference copy of the document with all sections, see nature.com/documents/nr-reporting-summary-flat.pdf

Life sciences study design

All studies must disclose on these points even when the disclosure is negative.

Sample size	No sample size was applied in this study to predetermine sample sizes for experiments using cell lines. A sample size of three was used to evaluate the spread of the data and was determined based upon other studies with similar methodologies (PMID: 26538579, PMID: 33188182).
Data exclusions	No data were excluded from the analyses.
Replication	All experiments were replicated as stated in the figure legends.
Randomization	Only biochemical and cell-based experiments (no animals) were carried out in our study and as such there was no need for the randomization of our samples.
Blinding	There was no blinding, as the same investigator performed most experiments and analyzed the data.

Reporting for specific materials, systems and methods

We require information from authors about some types of materials, experimental systems and methods used in many studies. Here, indicate whether each material, system or method listed is relevant to your study. If you are not sure if a list item applies to your research, read the appropriate section before selecting a response.

Materials & experimental systems

n/a	Involved in the study	
[ ]	[x]	Antibodies
[ ]	[x]	Eukaryotic cell lines
[x]	[ ]	Palaeontology and archaeology
[x]	[ ]	Animals and other organisms
[x]	[ ]	Human research participants
[x]	[ ]	Clinical data
[x]	[ ]	Dual use research of concern

Methods

n/a	Involved in the study	
[x]	[ ]	ChIP-seq
[x]	[ ]	Flow cytometry
[x]	[ ]	MRI-based neuroimaging

Antibodies

Antibodies used	anti-p44/42 MAPK (ERK1/2) (L34F12) Mouse mAb (Cell Signaling #4696; 1:3000 dilution; referred to as Anti-panERK or Anti-ERK antibody), anti-phospho-p44/42 MAPK (Thr202/Tyr204) Rabbit Ab (Cell Signaling #9101; 1:3000 dilution), anti-Flag (M2) Mouse mAb (Sigma F1804; 1:10000 dilution), anti-phospho-p90RSK (S380) (D3H11) Rabbit mAb (Cell Signaling #11989; 1:2000 dilution), Phospho-Akt Substrate (RXXS*/T*) (110B7E) Rabbit mAb (Cell Signaling #9614; 1:2000 dilution), Mouse Anti-alpha-Tubulin (Sigma #T6199; 1:10000 dilution)

	Secondary antibodies: IRDye 680 RD goat anti-Rabbit (Li-Cor #925-68071, 1:5000 dilution), IRDye 800 CW goat anti-Rabbit (Li-Cor #926-32211, 1:5000 dilution), IRDye 680 RD goat anti-Mouse (Li-Cor #926-68070, 1:10000 dilution), anti-rabbit IgG HRP-linked antibody (Cell Signaling #7074S, 1:10000 dilution), anti-mouse IgGRP-linked antibody (Millipore #401215, 1:10000 dilution)
Validation	The primary antibodies used in this study were all validated by the manufacturers and can be checked using their respective catalog # on the following websites:<br>Cell Signaling: https://www.cellsignal.com<br>Sigma: https://www.sigma.com

Eukaryotic cell lines

Policy information about cell lines

Cell line source(s)	HEK-293T (ATCC®, #CRL-3216TM), HEK293-ΔRSK1/2 cell line was described in Ref 18 (PMID:27099309)
Authentication	The cell line was not authenticated.
Mycoplasma contamination	The cell line was not tested for mycoplasma contamination.
Commonly misidentified lines (See ICLAC register)	Commonly misidentified cell line was not used in this study.
